# EPOPTIS: A Monitoring-as-a-Service Platform for Internet-of-Things Applications

**DOI:** 10.3390/s24072208

**Published:** 2024-03-29

**Authors:** Petros Zervoudakis, Nikolaos Karamolegkos, Eleftheria Plevridi, Pavlos Charalampidis, Alexandros Fragkiadakis

**Affiliations:** 1 Institute of Computer Science, Foundation for Research and Technology-Hellas (FORTH), GR70013 Heraklion, Greece; zervoudak@ics.forth.gr (P.Z.); nkaram@ics.forth.gr (N.K.); eleftheria@ics.forth.gr (E.P.); pcharala@ics.forth.gr (P.C.); 2 Department of Computer Science, University of Crete, GR70013 Heraklion, Greece

**Keywords:** internet of things, blockchain, smart contracts, data integrity, management, monitoring, FIWARE

## Abstract

The technology landscape has been dynamically reshaped by the rapid growth of the Internet of Things, introducing an era where everyday objects, equipped with smart sensors and connectivity, seamlessly interact to create intelligent ecosystems. IoT devices are highly heterogeneous in terms of software and hardware, and many of them are severely constrained. This heterogeneity and potentially constrained nature creates new challenges in terms of security, privacy, and data management. This work proposes a Monitoring-as-a-Service platform for both monitoring and management purposes, offering a comprehensive solution for collecting, storing, and processing monitoring data from heterogeneous IoT networks for the support of diverse IoT-based applications. To ensure a flexible and scalable solution, we leverage the FIWARE open-source framework, also incorporating blockchain and smart contract technologies to establish a robust integrity verification mechanism for aggregated monitoring and management data. Additionally, we apply automated workflows to filter and label the collected data systematically. Moreover, we provide thorough evaluation results in terms of CPU and RAM utilization and average service latency.

## 1. Introduction

In recent years, the technology landscape has been dynamically reshaped by the rapid growth of the Internet of Things (IoT), introducing an era where everyday objects, equipped with smart sensors and connectivity, seamlessly interact to create intelligent ecosystems. The evolution of the IoT has revolutionized how technology is perceived and utilized and opened the door for innovative and intelligent applications. From smart cities [1] and buildings [2], precision agriculture [3], and advanced energy management [4] to data acquisition and monitoring systems for hydrogen generators [5] and the bioleaching industry [6], the scope of IoT has expanded, fostering a connected world that leverages data-driven insights for enhanced efficiency; it is estimated (https://transformainsights.com/news/iot-market-24-billion-usd15-trillion-revenue-2030 (accessed on 25 March 2024)) that the number of IoT devices that will be in operation by 2030 will reach 21.1 billion, with a revenue of USD 1.5 trillion. Moreover, as Industry 4.0 combines traditional industries with cutting-edge technologies enabling smart processes and product realization, the IoT is a major driving force for efficient and massive decentralized data collection, supporting a complex system of diverse systems and devices [7].

The fundamental building blocks of the interconnected IoT network that makes up the IoT are smart devices and sensors that are equipped with smart communication abilities, make gathering and sharing data easy, and create a network of remarkable complexity, in this way necessitating the implementation of (semi-)automatic mechanisms to manage their diverse components effectively. Moreover, such networks often function in distant and challenging environments, making sensors susceptible to failures and malfunctions. Consequently, autonomous monitoring and maintenance are crucial to mitigate the risk of disruptions and ensure adherence to Service-Level Agreements between IoT application providers and consumers. Monitoring can follow either a passive or active approach, encompassing the collection and logging of data from different network subsystems. Data generated by modern IoT systems are typically stored in cloud storage services (CSSs) for subsequent processing and analysis tasks, including anomaly detection [8], fault diagnosis [9], predictive maintenance [10], and more. Utilizing external CSSs helps to address the difficulties associated with local storage management; however, it also introduces an increased risk of data manipulation on remote servers. Unfortunately, such tampering can reduce the precision and reliability of subsequent processing or analysis, compromising decision-making effectiveness; thus, data integrity becomes crucial for the efficient cloud storage of the collected data. IoT devices are highly heterogeneous in terms of software and hardware (https://datatracker.ietf.org/doc/html/rfc7228 (accessed on 25 March 2024)), and many of them are severely constrained (processor, memory, storage). This heterogeneity and potentially constrained nature creates new challenges in terms of security, privacy, and data management. Moreover, there is an increase in users’ privacy concerns [11,12] as personal identifiable information is collected by IoT devices (e.g., wearables) and stored in cloud locations users are not aware of.

There is a plethora of IoT platforms and protocols ([13]), with their scope falling in three main categories: (i) those used for pure data collection, (ii) those used for management purposes, and (iii) hybrid, used for data collection as well as for management. Regarding data management, the protocols used mainly split into two categories: (i) IoT data collection protocols and (ii) IoT network management protocols. IoT data collection refers to the protocols and the support mechanisms for collecting IoT sensory data, including everything from determining the type and format of the data to be collected and deciding how frequently data should be collected to the methods by which data are transmitted from devices to the network. Additionally, IoT data collection protocols also encompass the issue of commands for actuation purposes, such as controlling a valve based on the data collected from the sensors. Several protocols of this type have been developed, such as the COAP [14], LwM2M (https://omaspecworks.org/what-is-oma-specworks/iot/lightweight-m2m-lwm2m (accessed on 25 March 2024)), MQTT (https://mqtt.org (accessed on 25 March 2024)), etc. On the other hand, the IoT network management protocols oversee the status, configuration, and performance of individual IoT devices on the network, including tasks such as device provisioning, firmware updates, and error handling. They also focus on the overall health and performance of the network as a whole, including tasks such as traffic management, resource allocation, and security. Overall, effective data management protocols are essential for ensuring that IoT devices function properly and that IoT data are collected and processed in a secure and efficient manner. Popular protocols include SNMP [15], NETCONF [16], RESTCONF [17], CORECONF [18], etc.

This work proposes EPOPTIS (EPOPTIS is the Greek word for supervisor), a Monitoring-as-a-Service (MaaS) platform for both monitoring and management purposes, offering a comprehensive solution for collecting, storing, and processing monitoring data from heterogeneous IoT networks for the support of diverse IoT-based applications. To ensure a flexible and scalable solution, we leverage the FIWARE open-source framework (https://www.fiware.org (accessed on 25 March 2024)), also incorporating blockchain (BC) and smart contract (SC) technologies to establish a robust integrity verification mechanism for aggregated monitoring and management data. Additionally, we apply automated workflows to filter and label the collected data systematically. The existing literature on data integrity verification for IoT data stored in cloud storage predominantly relies on encryption techniques, often coupled with the trustworthiness of Third-Party Auditors (TPAs). In contrast, BC-based data integrity schemes offer a compelling alternative by eliminating the need for TPAs, thereby addressing the trust issue. However, they have to face the issues of significant storage overhead, especially when considering the direct storage of the raw data in a BC ledger. To address the challenges mentioned, our solution adopts a strategic approach that involves the handling of raw IoT data in distinct time-windows. For integrity verification purposes, we generate a set of verified tags that are stored in the BC ledger. This methodology is designed with the goal to minimize the cost associated with data storage. Our main contributions are summarized as follows: (i) we propose a Monitoring-as-a-Service platform for IoT applications based on FIWARE, (ii) we utilize BC and SC technologies for data integrity verification purposes, and (iii) we thoroughly evaluate the platform in terms of CPU and RAM utilization and average service latency.

The remainder of the paper is organized as follows. Section 2 provides a summary of related works. In Section 3, we describe the system requirements and logical architecture of the proposed platform. In Section 4, the implementation details of the platform are presented. In Section 5, we present and discuss the performance evaluation results, and, finally, the conclusions and further work appear in Section 6.

## 2. Related Works

This section presents works related to the proposed platform contributions, which mainly consist of (i) generic platforms for the support of IoT applications that do not utilize BC and SC technologies and (ii) platforms that use BC technology to accomplish automatic data integrity verification.

The authors in [19] propose an IoT platform based on edge computing to support various types of devices located within cities, introducing a cloud-native environment to solve operation and maintenance management problems. The proposed platform is divided into three layers: (i) a Terminal Device Layer, (ii) an Edge Layer, and (iii) a Cloud Layer, performing tasks such as data collection, pre-processing and partial storage of the incoming data, etc. The authors also describe a Smooth Weight Round-Robin algorithm for load balancing. Nevertheless, no evaluation results or details on various fundamental components of the platform (i.e., identity management and access control) are provided. Article [20] proposes MEWiN, a platform for precision agriculture that is based on FIWARE, using components such as the Cosmos Generic Enabler, Orion Context Broker, etc.; however, the evaluation results presented are limited to the power consumption of the devices, battery voltage discharge, and specific types of data collection (e.g., water content values). A Cloud-IoT-based sensing service for health monitoring is presented in [21], which consists of various layers (data collection, data management, application service), supporting wearables and smart devices. In [22], the authors propose a FIWARE-based platform for remote patient monitoring, considering various users such as physicians, medical operators, and patients. The platform consists of three logic layers: (i) a Front-end Layer that includes all required components for the interaction with the medical and paramedical staff, (ii) an Elaboration Layer, which implements the functionalities for health data gathering and storage, and (iii) a Security Layer that provides access control and identity management; however, no performance evaluation results are provided. The authors in [23] propose a FIWARE-based platform for sensor data monitoring in seaports, employing various FIWARE components, such as the Orion Context Broker, Cosmos, etc. The collection of various types of data, such as the wave height, ship orientation, etc., is demonstrated but without any evaluation results to showcase the scalability of the platform. The authors in [24] describe a monitoring platform for energy management that consists of three layers (acquisition, transmission, management), using the RS-485 and MODBUS-RTU protocols for the data communication between the acquisition devices and the data center services. OpenHab [25] is a freeware IoT platform that implements an open-source solution to the Eclipse SmartHome framework, using Apache Karaf and Eclipse Equinox runtime [13]. SmartThings [26] is a proprietary home automation platform developed by Samsung that follows the producer/consumer paradigm, supporting sensors and actuators. There are various other commercial platforms, such as the Apple HomeKit [27], Amazon Web Services IoT [28], IBM Watson [29], etc., which require commercial agreements or subscriptions. All of the aforementioned platforms and services have two common characteristics: (i) their core operations are centralized and hence threats that emerge as single-point of failures are possible (also considering the increased number of cyber-attacks worldwide) (https://www.enisa.europa.eu/publications/enisa-threat-landscape-2023 (accessed on 25 March 2024)), and (ii) they are vertical implementations having a very narrow scope, servicing only specific applications (e.g., healthcare).

Several other contributions use BC technology to accomplish automatic data integrity verification. Article [30] presents a BC-based scheme where integrity verification is performed by SCs; however, there are several limitations: (i) this is a standalone service that has not been evaluated within a complete IoT platform, (ii) third parties need to use a BC-compliant client in order to communicate with the BC, (iii) fees (Gas) have to be paid for the services as the Ethereum (https://ethereum.org (accessed on 25 March 2024)) BC is used, and (iv) there is a significant overhead for data encryption/decryption as the (hashed and encrypted) data are stored in the public ledger. The authors in [31] propose a data integrity detection model based on BC, with a process divided into three parts: data generation, data storage to the BC, and data fetching from the BC. In this work, data are directly stored in the BC, which is not efficient, and the evaluation is performed in a simulated environment that is not realistic given the current advances in BC technology. The HyperLedger Fabric (https://www.hyperledger.org/projects/fabric (accessed on 25 March 2024)) BC (HLF) is used in [32] to provide data integrity and storage, also employing IPFS (https://ipfs.tech/distributedstoragesystem (accessed on 25 March 2024)) for distributed storage. This platform consists of four layers (physical, network, middle, application), and the authors have demonstrated its scalability in terms of throughput and transaction time and average latency; however, a limitation is that the integrity verification process is “hardcoded”, merely based on BC and HLF, as no SCs are employed. The authors in [33] propose a BC-based data verification scheme that splits into three stages (setup, processing, verification), using bilinear mapping techniques; however, their solution has two weaknesses: (i) various cryptographic operations (e.g., digital signatures) are required by clients that collect data, so it may be infeasible for this scheme to support constrained IoT devices, and (ii) the BC technology used is not based on a real BC such as HLF, Ethereum, etc., and rather it has been simulated using the Python programming language, so its robustness and usage in real environments is questionable. In [34], the authors present an integrity verification scheme that uses three types of SCs for: (i) checking data owners’ legitimacy (using the RSA algorithm), (ii) checking data repository’s (cloud servers) reliability using Merkle hash tree, and (iii) preventing replay attacks using bilinear mapping. Here, a different approach (as compared to EPOPTIS) is taken as only the data owners can request their data, so support for third-party applications (e.g., within a smart city context) cannot be easily supported. The authors have evaluated SC execution in an Ethereum network, but it is not clear if the core functionalities of the scheme can execute on Ethereum. The authors in [35] present a BC-based data integrity verification scheme for smart home applications defining five basic components: smart devices, trusted third parties, cloud service providers, home gateways, and blockchains. They utilize the home gateway to aggregate all data information and formulate homomorphic verifiable tags for verification purposes. However, there are several limitations: (i) limited evaluation results are provided, (ii) as a private bitcoin network is used, it cannot support a high number of transactions due to the Proof-of-Work consensus algorithm used, (iii) no flexibility is provided as SCs are not used, and (iv) the scheme is not integrated or tested within a real platform. Finally in [36], the authors propose a platform for smart city applications that is based on two BC levels: (i) private BCs that store the IoT data provided by the various city organizations (e.g., water management, energy management, etc.) and (ii) a consortium BC that stores the IoT data provided by the private BCs. The drawbacks of this approach are that multiple consensus algorithms have to execute prior to persistent IoT data storage, thus complexity and latency increase, and IoT devices have to send their data through BC transactions that may not be feasible if the devices are constrained (in terms of memory and processing). The related contributions that do not utilize BC technology for data integrity verification are shown in Table 1, while those that do utilize it are summarized in Table 2, along with their limitations.

## 3. System Requirements and Logical Architecture

This section presents the system requirements and the logical architecture of the EPOPTIS platform.

### 3.1. System Requirements

Following the common convention, system requirements are categorized into two types: *functional* and *non-functional*. *Functional* requirements define specific functions and capabilities that the platform must possess to meet the needs of its users, while the *non-functional* ones address constraints and performance characteristics that the system must exhibit to ensure its overall quality and user experience.

#### 3.1.1. Functional Requirements

- **Heterogeneous device support:** The platform should consider the inherent heterogeneity of native IoT device hardware platforms and ensure seamless integration, offering flexible enrollment for various device flavors.
- **Device virtualization:** Physical devices and their resources should be appropriately virtualized, adhering to a standardized information model that allows easy querying though common syntax.
- **Ubiquitous access and transparent communication:** Devices should be accessible irrespective of constraints imposed by end-node network topology and configuration. In scenarios where devices do not support IPv6 or are not IPv6 routable, devices should be accessed though transparent gateways that provide protocol translation or Network Address Translation.
- **Multi-tenancy:** Multi-tenancy mechanisms should be supported, providing complete resource isolation of different tenants and preventing unauthorized access.
- **Data processing of large-scale monitoring data:** The platform should be able to process and analyze large amounts of monitoring data and provide useful insights based on user-defined metrics.
- **Visualization of monitoring data:** The platform should visualize monitoring data, data integrity verification, and event-based notifications in a comprehensible and user-friendly manner (e.g., graphs, labels, etc.).
- **Data integrity:** The platform should ensure the integrity and consistency of monitoring data throughout its life cycle by developing robust mechanisms to prevent unauthorized modifications. This enhances the reliability and trustworthiness of the monitoring data, maintaining its accuracy and validity when visualized.
- **Real-time alerting:** A robust event-based notification system that operates on predefined rules should be incorporated, promptly alerting users about the violations of these rules. The platform should be capable of generating real-time alerts, ensuring timely communication of critical events or sensory data deviations from predefined rules.

#### 3.1.2. Non-Functional Requirements

- **Security and privacy:** The platform should integrate robust authentication and authorization mechanisms for users and IoT devices. Control and application data communication should be encrypted and integrity-protected.
- **High availability:** The platform should provide high availability of monitoring services as well as an IoT data storage service, taking into consideration diverse network conditions.
- **Fault tolerance:** The platform should be resilient and able to recover from faults and failures on both cloud and IoT devices.
- **Scalability:** The platform should be capable of handling large volumes of data in terms of storage, retrieval, and processing capabilities.
- **Quality of service (QoS):** QoS should be maintained as high as possible in terms of interactions with cloud services (e.g., low latency of data retrieval, near-real-time notification mechanism) and IoT devices (e.g., low latency of IoT data updates).

### 3.2. Logical Architecture

**Figure 1:**
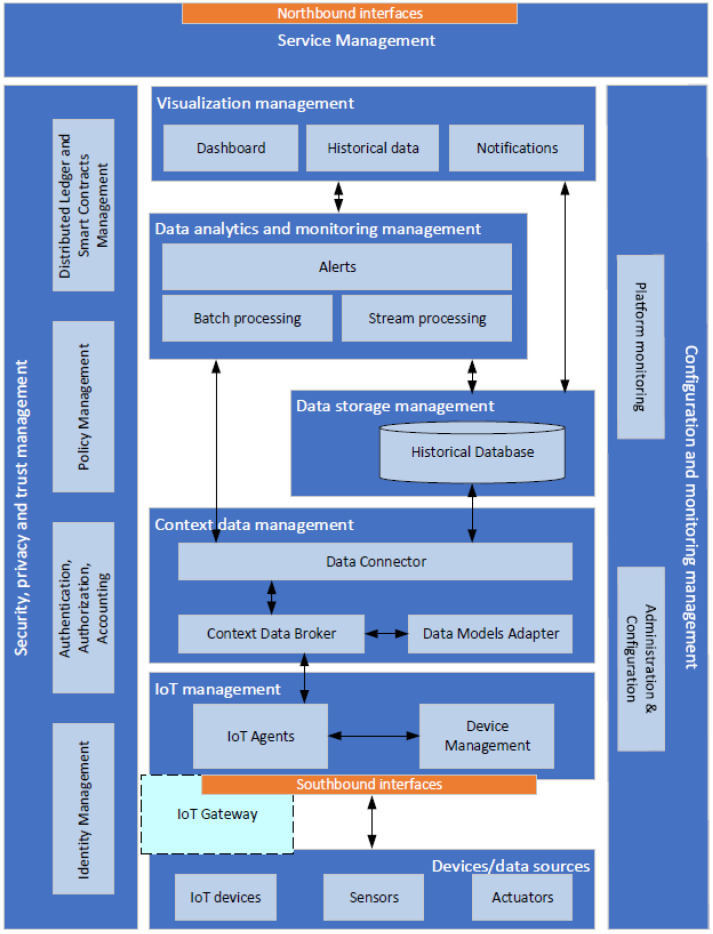
Logical architecture of the EPOPTIS platform.

Here, we present the logical architecture of the proposed MaaS platform, with its components shown in Figure 1.

The *service management* layer provides appropriate application programming interfaces (APIs) for the interaction of external applications/users with the services offered by the platform. It acts as the main endpoint and service orchestrator, handling incoming requests and discovering and orchestrating all the internal services.

The *visualization management* layer provides various mechanisms for visualizing context information pertaining to IoT networks managed by the platform. It encompasses a variety of functions, including the integration of IoT network status and measurements in a user-friendly dashboard. Additionally, it provides charts that showcase historical IoT sensory data and its integrity validity. Moreover, it presents notifications generated by the platform, ensuring that users are promptly informed about critical events and updates.

The *data analytics and monitoring management* layer provides a robust mechanism for processing the monitoring data collected from the IoT networks. The primary goal of this layer is to provide valuable insights into network performance and the sensory data collected. Additionally, it supports event-based notifications in response to abnormal situations or operation errors. Data processing can be performed either (near) real-time, as context data are collected, or on historical data persistently stored by the platform.

The *data storage management* layer provides essential functionalities for the secure, reliable, and persistent storage of context information originating from heterogeneous IoT networks.

The *context data management* layer is responsible for managing context information collected from IoT networks, offering a unified and standardized interface to access this information. Generally, contextual information is defined as the current state of all entities across the platform and it is represented in a structured manner using appropriate data models.

The *IoT management* layer is an entity responsible for bridging the communication gap between IoT devices and the *context data management* layer. Its primary goal is to ensure the compatibility and availability of these devices on the platform by implementing suitable protocol/data model translators. Additionally, this entity facilitates the management of heterogeneous IoT devices, encompassing functionalities such as registration, configuration, and monitoring.

The *devices/data sources* layer consists of IoT devices, including end devices equipped with sensors and/or actuators that enable the monitoring of their operational environment.

The *security, privacy, and trust management* layer is a cross-layer entity responsible for all operations, policies, and mechanisms implemented to ensure security, privacy, and trust in the platform’s pillars (i.e., the cloud services of the platform and the IoT networks). This entity offers critical security and trust mechanisms for user authentication, authorization, access control, secure device bootstrapping, and data integrity verification. Security and system robustness is provided by utilizing the BC and SC technologies as (i) the data are stored in the immutable ledger (actually stored in replicas in multiple nodes), and hence data corruption is not feasible as it would require the corruption of the data in all possible locations and the re-calculation of the cryptographic hashes of all previous block stores in the ledger; (ii) the consensus algorithm executed by the BC peers guarantees that no malicious or faulty transactions can be validated within the BC network as long as the majority of the peers are properly functioning; (iii) SC states are protected through the immutable ledger and the consensus algorithm and their outputs are valid as long as the majority of the peers are honest; and (iv) the data producers (e.g., IoT devices) are authenticated and authorized by utilizing suitable authorization tokens and FIWARE components such as the Keyrock, while their data are encrypted in transit using transport layer security (TLS). Privacy preservation can become feasible through the use of ephemeral bearer authentication tokens that do not reveal user/device identities; moreover, only the hashed values of the collected data are stored in the ledger, and thus the actual data are protected in case a compromised BC peer is present.

The *configuration and monitoring management* layer is also a cross-layer entity responsible for managing and monitoring the configuration parameters of the platform and its components. This entity allows the definition of specific Key Performance Indicators to evaluate a platform’s quality of services, reliability, and availability. By managing the configuration and continuously monitoring parameters, it ensures the system operates optimally and detects any deviations from normal conditions.

## 4. Implementation Details of the EPOPTIS Platform

In this section, we detail the system architecture of the proposed MaaS platform, initially, by defining the data model that describes the contextual information and then presenting its functional architecture and discussing the functional entities and their interactions.

### 4.1. Context Information Model

**Figure 2:**
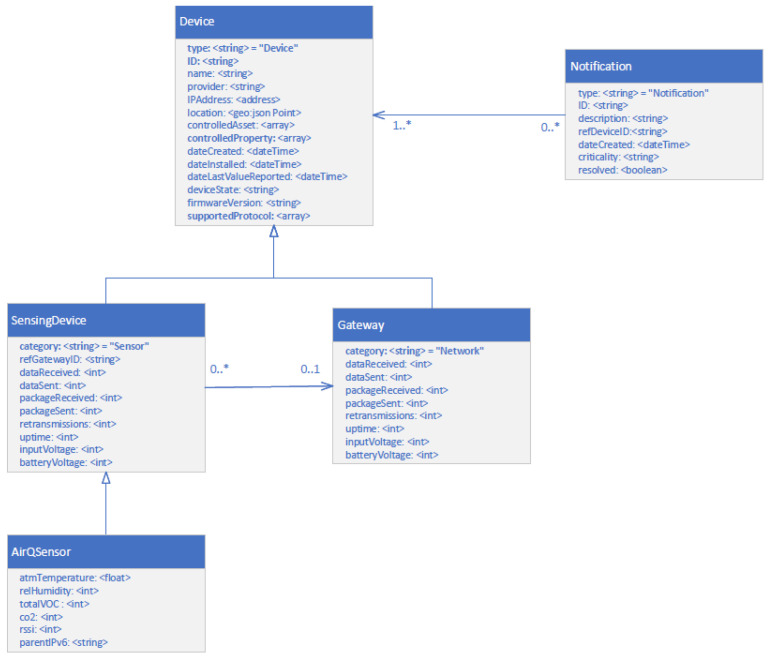
Contextual entities and their associations.

The *Context Information Model* (CIM) establishes a standardized and structured representation of contextual information, facilitating the capture, organization, and sharing of such information between IoT networks and the monitoring platform. Within the CIM, context information is represented using entities, attributes, and relationships. Entities can correspond to conceptual abstractions or physical objects, while attributes describe the properties or characteristics of the entity. The relationships in this model define the semantic associations and dependencies between different entities. A CIM representation is illustrated in Figure 2, appropriately adjusted to align with the NGSI (Next-Generation Service Interface) protocol (https://fiware.github.io/specifications/ngsiv2/stable (accessed on 25 March 2024)). This adjustment ensures that the platform’s entities can efficiently communicate and integrate context information, fostering a harmonious and interconnected monitoring ecosystem. At the core of this data model, the *Device* entity acts as a fundamental abstraction, representing a generic device that encompasses essential attributes common to all devices. Depending on the nature of the physical devices, the *Device* entity can be specialized into two other distinct entities, each one tailored to specific characteristics. The *Sensing Device* entity represents specialization in sensor-related characteristics, facilitating the representation of data measurements. Additionally, further specialization for the *Sensing Device* entity provides a specific representation of air quality measurements, as defined in the *AirQSensor* entity. On the other hand, the *Gateway* entity specializes as a *Device*, representing all the characteristics required for a networking device. In this context, each *Device* entity can be associated with 0 to *n Notification* entities, indicating that a *Device* can have multiple *Notifications* related to it. The association between *Devices* and *Notifications* enables the effective monitoring and alerting within the platform, allowing users to receive timely and relevant information about potential issues or critical events associated with specific *Devices*.

### 4.2. Functional Architecture

**Figure 3:**
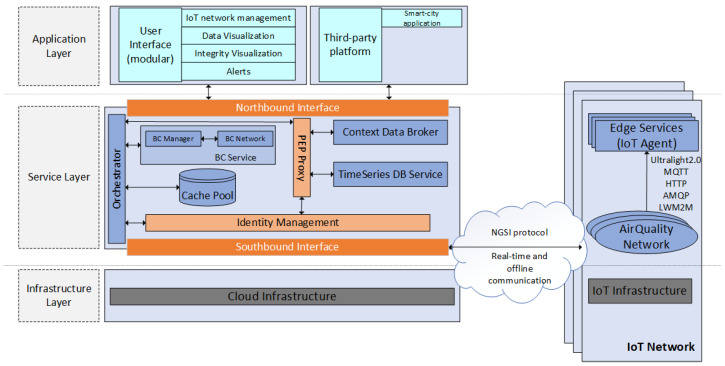
Functional architecture of the EPOPTIS platform.

In Section 3, we described the logical architecture of the proposed platform (Figure 1), while, in this section, we present its functional architecture, which comprises three layers, as depicted in Figure 3. The *Application Layer* encompasses user interfaces and dashboards used for visualizing all information collected by the MaaS platform. This layer can also feed third-party visualization platforms such as Kibana (https://www.elastic.co/kibana (accessed on 25 March 2024)), Grafana (https://grafana.com (accessed on 25 March 2024)), ThingSpeak (https://thingspeak.com (accessed on 25 March 2024)), etc. Currently, EPOPTIS feeds data with the visualization platform presented in [37].

The second layer, the *Service Layer*, involves various processes that provide critical functionalities, including identity and authorization management, contextual information management, historical data management, data integrity verification, and alert-based notification mechanisms. In this regard, FIWARE offers a set of specifications accessible through well-defined interfaces and supports a flexible architecture that facilitates the interconnection of devices with IoT applications. FIWARE’s adoption fits our platform’s needs as it simplifies development by providing a collection of extensible, scalable, and configurable components that can foster application development.

The third layer, the *Infrastructure Layer*, offers the necessary hardware and virtualized resources required to deploy the MaaS platform effectively. By adhering to the requirements as detailed in Section 3.1, the proposed architecture allows cloud services to interact seamlessly with IoT Devices, ensuring a robust and efficient MaaS platform.

#### 4.2.1. Orchestrator

In order to construct a reliable, robust, and secure cloud-based platform, the monitoring, management, and orchestration of a variety of underlying heterogeneous technologies is required. In the core of the proposed architecture resides the *Orchestrator*, which serves as a gateway between our platform and third-party applications or IoT networks that wish to utilize the underlying provided services. The *Orchestrator* receives and verifies submitted requests to the platform, performs the corresponding actions, and finally generates the appropriate responses. It offers a RESTful API for communication and data exchange, consisting of two logically separated interfaces: (a) the *Northbound Interface*, which handles requests from user interfaces and third-party platforms, enabling the data retrieval of monitoring data and other provided services, and (b) the *Southbound Interface*, which handles requests from the IoT networks. The *Orchestrator* implements a number of software modules, named *Resource Modules*, which are responsible for managing the various heterogeneous system resources. Upon receiving a request, it communicates with the internal services, as dictated by the processing workflow, and finally returns the appropriate response. Below, we provide a description of the *Resource Modules*:

- ***Identity Module:*** This module is responsible for managing information about logical hierarchical entities on our platform, including virtual representation of human and non-human users (i.e., IoT users), ecosystems, roles, permissions, and their relevant connection graphs. The *Identity Module* utilizes the *Identity Management* component to store and associate this type of information in its internal database.
- ***Device Module:*** This module is responsible for retrieving information regarding physical devices and their association with IoT networks, supporting the retrieval of essential information about both types of physical devices, gateways, and sensing devices, including their attributes, as described in the contextual model. The *Device Module* utilizes the *Context Data Broker* component to retrieve this kind of information.
- ***Statistics Module:*** This module is responsible for retrieving and calculating statistics about logical entities and their associations. It provides detailed information about users, devices, and ecosystems, including statistics such as the number of users within an IoT ecosystem and the assigned role for each user within an IoT network. The *Statistics Module* utilizes the *Context Data Broker* and the *Identity Management* components to provide this type of information.
- ***Notification Module:*** This module is responsible for the retrieval of information regarding generating notifications and organizing them in two categories: the *IoT Ecosystem* or the *Device*. The retrieved information includes criticality level, descriptions, and the attribute on which the notification was triggered. Additionally, this module allows authorized users to manage generated notifications (e.g., appropriately labeling them once they have been resolved). The *Notification Module* utilizes the *Context Data Broker* to support these operations.
- ***IoT Module:*** This module is responsible for registering a new device and updating its attributes. It receives NGSI payloads from IoT networks and implements mechanisms for: (i) triggering alert-based notifications according to the SC rules, (ii) caching the incoming NGSI payloads using a *Cache Pool*, calculating and storing the appropriate hash value required for the data integrity verification, and (iii) updating contextual information in order to support a seamless interaction with the IoT networks. This module utilizes the *Cache Pool*, the *Context Data Broker*, and the *BC Service* (described in Section 4.2.6) in order to support all the above operations.
- ***Query Module:*** This module is responsible for retrieving historical data and labeling them utilizing the data integrity verification mechanism. A detailed description of how this mechanism works is provided in Section 4.3. This module utilizes the *TimeSeries DB* service for retrieving historical data and the *BC Service* to obtain the appropriate hash value used to label them according to the outcome of the integrity protection mechanism (i.e., corrupted/not corrupted).

#### 4.2.2. Identity Management and PEP Proxy

These two components are responsible for the authentication and authorization operations of the platform, which encompass: (i) identity and user management and (ii) user authorization and access control. The *Identity Management* component provides the ability to create, update, and manage user accounts, storing user profile information, authentication credentials, roles, and permissions. When a request is made to access a protected resource, the *PEP Proxy* intercepts it and enforces the access control policies defined in the *Identity Management* component. Here, we utilize the FIWARE Keyrock (https://fiware-idm.readthedocs.io/en/latest (accessed on 25 March 2024)) for identity and user management and the Wilma PEP Proxy (https://fiware-pep-proxy.readthedocs.io/en/latest (accessed on 25 March 2024)) for authorization and access control.

#### 4.2.3. Cache Pool

This component acts as in-memory data storage for the NGSI payloads, which are grouped within specific time intervals before being fed into the caching mechanism. A detailed description of how this mechanism works is provided in Section 4.3. Here, we utilize Redis (https://github.com/redis/redis (accessed on 25 March 2024)) for the in-memory data storage.

#### 4.2.4. Context Data Broker

When connecting IoT devices to an IoT platform, the publish/subscribe pattern has proven to be a suitable method for messaging [38]. To this direction, the *Context Data Broker* component achieves the decoupling of producers and consumers of context information, implementing the Publish/Subscribe design pattern based on the FIWARE Orion Context Broker (https://fiware-orion.readthedocs.io/en/master (accessed on 25 March 2024)). Context producers (e.g., IoT ecosystems) publish their data to this component through the FIWARE NGSI API, without the requirement to know who the consumers of such data are. Context consumers (e.g., third-party applications, historical databases) do not need to know the origin of the data but are solely interested in the event itself which is consuming them. This decoupling mechanism allows context-aware IoT ecosystems to interact with the context information in a flexible and scalable manner, enabling seamless integration and fostering the development of a context-driven approach. The *Context Data Broker* supports the querying of the last value referring to the attributes of each context entity and also provides a subscription mechanism, enabling the persistent storage of the historical attributes.

#### 4.2.5. TimeSeries DB Service

This is a component based on FIWARE QuantumLeap (https://quantumleap.readthedocs.io/en/latest (accessed on 25 March 2024)), designed to provide persistent storage of context information from the *Context Data Broker* in an external repository by converting the NGSI structured data into a tabular format and persistently storing context changes in a high-performance CrateDB database (https://crate.io (accessed on 25 March 2024)). It also provides an interface for performing complex queries on historical data (e.g., the latest *N* samples collected from a specific IoT device, the minimum value over a time period per hour or month, etc.).

#### 4.2.6. Blockchain Service

Data integrity verification is one of the core functionalities of the proposed platform and this is utilized through the *BC Service*, which consists of two discrete components, namely (i) the *BC Network* and (ii) the *BC Manager*. The *BC Network* maintains a permissioned decentralized ledger based on HLF that facilitates the process of transaction recording and asset tracking in an immutable manner. The network structure and data flow are designed and implemented in order to achieve high service availability and to minimize the risk of being compromised. The utilization of a private HLF network offers several key advantages, particularly in terms of high availability and reliability. Using HLF, we have utilized a network of four organizations (https://hyperledger-fabric.readthedocs.io/en/latest/network/network.html (accessed on 25 March 2024)), three acting as *Peer* organizations and one as *Orderer*. Each of the *Peer* organizations operates with two *Peer* nodes, while the *Orderer* organization uses two *Orderer* nodes. The endorsement policy used assures a high availability, also requiring the majority of organizations to endorse transactions using at least one of their *Peer* nodes. This redundancy ensures that if a *Peer* node experiences downtime for any reason, the other *Peer* nodes within the same organization can seamlessly take over during transaction validation. Similarly, the presence of two *Orderer* nodes within the *Orderer* organization, guarantees fault tolerance in case a node becomes unavailable.

The *BC Manager* functions as a client for one of the three peer organizations. A client in the context of HLF refers to an authorized application that can interact with the *BC Network*, essentially acting as an interface (implemented as a REST API with Golang) that links the HLF network with the *Orchestrator*.

The *BC Service* provides two core functionalities:

- ***Data labeling***. SCs are crucial for the automated label compliance assignment of the collected IoT data. These enforce configurable rules that encompass various criteria, such as acceptable sensor data ranges, input voltage limits, etc. When data are submitted to the platform, SCs automatically assign a label based on these predefined criteria. Therefore, during the data retrieval processes, the historical data are labeled as compliant or non-compliant (based on the previously reported rules), also feeding a suitable API for visualization purposes.
- ***Hash computation and storage***: The *BC Service* is responsible for storing and retrieving hash values, which are computed by the *Orchestrator* using the SHA-3 (https://csrc.nist.gov/pubs/fips/202/final (accessed on 25 March 2024)) hash algorithm. The corresponding hashed values are securely stored in the immutable BC ledger in a key-value format, significantly reducing retrieval times and conserving storage space within the ledger, while the actual data records are stored in the *TimeSeries DB*. The hashed values can be retrieved by the *Orchestrator* in order to support the data integrity verification mechanism during historical data retrieval.

Overall, the *BC Service*, with its decentralized nodes, automated recovery mechanisms, and data validation and storage processes, offers a resilient and reliable foundation for managing and securing IoT data.

### 4.3. Interactions of the Functional Components

#### 4.3.1. NGSI Data Persistent Storage

Prior to sending an NGSI payload to the *Orchestrator*, the *IoT Agent* (Figure 1) authenticates and obtains an access token with permissions that allow access to the platform’s internal services. In general, an NGSI request can be of two types: (i) a registration request, indicating that the NGSI payload is associated with a device that has not been already defined in the contextual entities (Figure 2), or (ii) an update request, signifying that the NGSI payload should update an existing contextual entity. After receiving an NGSI request from an *IoT Agent*, the *Orchestrator* manages and orchestrates all internal services for the (i) creation of the appropriate label for the payload; (ii) update of the relevant *Notification* entity to the *Context Data Broker*, if required; (iii) update of the relevant *Device* entity to the *Context Data Broker*; (iv) trigger of the appropriate broker notification to store the related attributes persistently in the *TimeSeries DB*; and (v) support of the mechanism that caches the incoming payloads and computes the corresponding hash values that are finally stored in the BC ledger.

**Figure 4:**
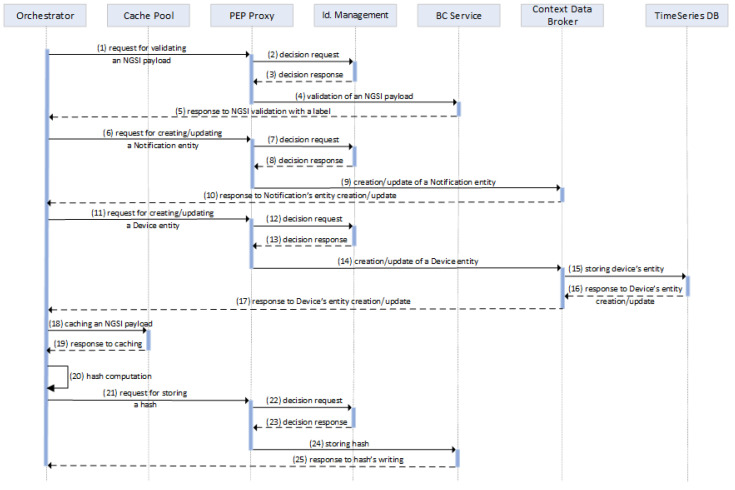
Functional component interactions for storing NGSI data.

In more detail and as shown in Figure 4, the *Orchestrator* sends a request for the validation of an NGSI payload (1). The *PEP Proxy* monitors the request flow and checks whether or not the request has the permission to access the *BC Service*’s resources for validating the payload’s content using the SC-based rules. The *Identity Management* service acts as the policy decision point (PDP) and infers if the request should access the specific resource or not (2, 3). The *PEP Proxy* enforces PDP’s decision to the *BC Service*, enabling or not the validation of the NGSI payload (4). The *BC Service* responds with an appropriate label that characterizes the payload’s validity (5).

Subsequently, if the payload does not validate the SC-based rules, the *Orchestrator* creates (or updates) the appropriate *Notification* entity in the *Context Data Broker*. The *PEP Proxy* monitors the request flow and checks whether or not the request has the access permission to create or update a *Notification* entity (6) and the PDP infers if the request has the permission to access the specific resource or not (7, 8). The *PEP Proxy* enforces PDP’s decision to *Context Data Broker*, enabling or not the creation or update of the *Notification* entity (9). Once the *Notification* entity is created or updated, the *Context Data Broker* stores the latest value of a notification and then sends the *Orchestrator* a suitable response message (10).

Additionally, the *Orchestrator* creates (or updates) the *Device* entity (11), ensuring that the contextual entities are kept updated to the latest state. The *PEP Proxy* enforces access control to the *Context Data Broker* (12, 13), triggering the appropriate contextual notifications (14), persistently storing the historical attributes of the *Device* entity (15). Once the payload’s attributes are successfully stored in the *TimeSeries DB*, the appropriate responses are generated (16, 17). Moreover, the *Orchestrator* implements a caching mechanism that facilitates the calculation of the hash value that for a group of payloads. Upon receiving NGSI payloads, the *Orchestrator* caches them in the *Cache Pool* (18) and awaits the appropriate response (19). Once a predefined volume of payloads has been cached, the *Orchestrator* calculates the hash value of this payload group (20) and stores it in the BC ledger (24). The access to the *BC Service* requires the access control process enforced by the *PEP Proxy* on the *BC Service*’s resources (21, 22, 23). The hash value is generated using the SHA-3 secure hash algorithm, enabling the immutable storage of an imprint of the data instead of storing the actual data themselves. This approach provides a more efficient and secure way to represent the data in the *BC Service*, reducing storage requirements and ensuring data integrity.

#### 4.3.2. Retrieval of Historical Data

**Figure 5:**
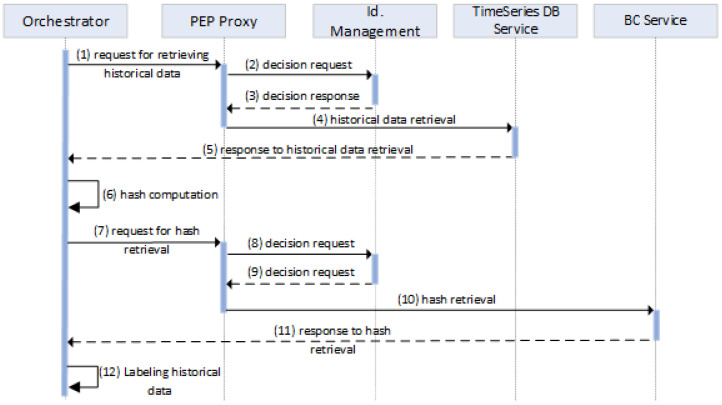
Functional component interactions for retrieving historical data.

After receiving a retrieval request, the *Orchestrator* manages and orchestrates all internal services for (i) retrieving historical data from the *TimeSeries DB*, (ii) calculating the hash value of the retrieved data, (iii) fetching the hash value through the *BC Service* (which was previously computed during storage), and (iv) comparing the hashes and labeling the retrieved data, inferring its integrity. All these interactions are depicted in Figure 5. Initially, the *Orchestrator* sends a request in order to retrieve historical data (1). The *PEP Proxy* checks if access to the resources of the *TimeSeries DB Service* for retrieving the data is permitted. The *Identity Management* service acts as the PDP and infers if the request should access the specific resource or not (2,3). The *PEP Proxy* enforces the PDP’s decision to the *TimeSeries DB Service*, enabling or not the retrieval of the historical data (4). The *TimeSeries DB Service* provides the requested data (5), and then, the *Orchestrator* calculates the corresponding hash value (6). Additionally, the *Orchestrator* retrieves the hash value that was previously stored by sending a request to the *BC Service* (7). The access to this service requires the access control process enforced by the *PEP Proxy* on the *BC Service*’s resources (8, 9, 10). The *BC Service* responds with the hash value that was previously stored (11). By comparing this with the hash value calculated for the recently retrieved data (12), any (unauthorized) modifications of the initial data received from *Orchestrator* during the storing process can be detected.

## 5. Performance Evaluation

With the intended goal to act as a multi-tenant solution offering unified monitoring and management services for heterogeneous IoT networks, the cloud platform presented in this work needs to efficiently handle a substantially large number of IoT devices pushing data updates, either directly or through appropriate gateways. Therefore, in this section, we provide an extensive performance evaluation of a Proof-of-Concept (PoC) deployment of the proposed platform, under variable workload, and further present and discuss the results of the evaluation.

### 5.1. Testbed and Deployment

Our testbed essentially comprises two distinct entities, namely (i) the *Load Generator infrastructure* and (ii) the *Cloud Infrastructure*. The *Load Generator* (LG) is responsible for generating configurable workloads (IoT device data updates) for the cloud backend and collecting appropriate performance metrics. We used *Apache JMeter* (https://jmeter.apache.org (accessed on 25 March 2024)) as the specialized software for workload generation and performance metric collection, which is a mature and industry-grade modular open-source tool used for the automated performance evaluation of web-based systems through load and regression tests. Developed in Java, it integrates support for various protocols and applications (e.g., HTTP(s), REST Webservices, FTP, LDAP, etc.) and supports the creation and execution of configurable and complex workloads on a web server, collecting and storing replies, as well as analyzing and presenting aggregate test statistics (e.g., latency, throughput, response time, etc.).

On the other hand, the *Cloud Infrastructure* (CI) hosts a complete deployment of our platform, as depicted in Figure 3. The cloud backend leverages several Generic Enablers of the FIWARE ecosystem, as described in Section 4.2. Specifically, the *Orchestrator* is a web application developed in Python, which is responsible for orchestrating the rest of the backend services and exposing a unified and comprehensive RESTful API for third-party applications to consume. Additionally, we developed the *BC Manager*, named here as *Pearl*, which is responsible for the deployment and maintenance of the *BC Service*, necessary for the IoT data validation and integrity verification functionalities offered by the platform. Essentially, it is an application written in the Go programming language that offers a rich RESTful API for the interaction between the *Orchestrator* and the *BC Service*.

Moreover, we used a microservices architecture [39] for the cloud-based deployment of the platform. In particular, we employed Docker containers operated by the Container Orchestration Engine, Kubernetes (https://kubernetes.io (accessed on 25 March 2024)), which conveniently enables the management of distributed and horizontally scalable cloud-native applications, with an emphasis on high availability and fault tolerance. We deployed Kubernetes in a self-managed private cluster that consists of six virtual machines (VMs): one master node and five worker nodes, one of which exclusively hosts the *BC Service*, i.e., both the *BC Manager* and the *BC Network*. Table 3 summarizes the VM specifications used as LG infrastructure and CI infrastructure for the platform deployment.

From a performance point of view, we enabled CPU pinning (tying virtual CPUs to real physical CPUs of the host) for the VM hosting the CI, with KVM hypervisor set in “host-passthrough” CPU mode. Preliminary experimentation showed that this configuration offers substantial performance gains compared to different configuration choices. In addition, we tuned the operating system limits so that our measurements reflect the capabilities of the applications, instead of the limits enforced by the operating system. Thus, the user limits for file size, max memory, and CPU time were set to unlimited, and the open file limit was increased to 64,000.

**Figure 6:**
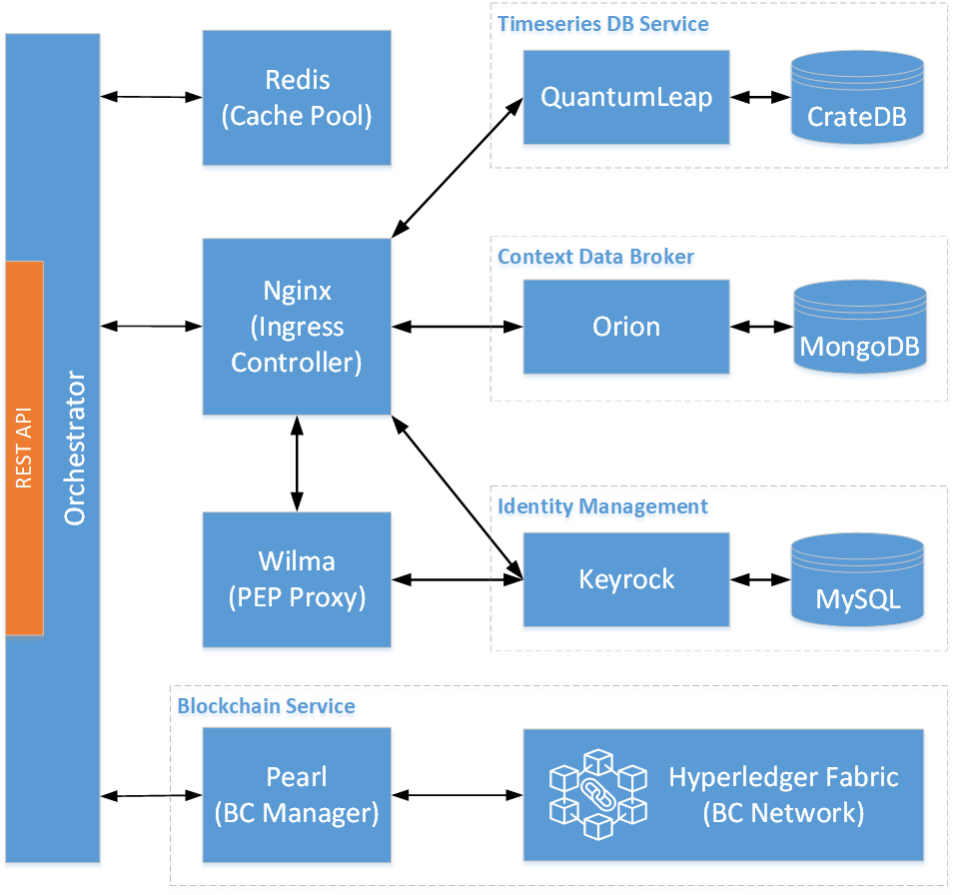
Interactions between the deployed platform components.

We used the *MicroK8s* (https://microk8s.io (accessed on 25 March 2024)) distribution for installing the Kubernetes cluster, which is maintained by Canonical (https://canonical.com (accessed on 25 March 2024)) and is provided through the *snap* package manager. It offers a lightweight *Certified Kubernetes Software Conformance* (https://www.cncf.io/training/certification/software-conformance (accessed on 25 March 2024)) distribution, which simplifies the selection of several Kubernetes functionalities through easy (de-)activation of add-ons (e.g., DNS, ingress, metrics-server, etc.). In addition, we utilized the *Helm* (https://helm.sh (accessed on 25 March 2024)) package manager for installing and maintaining the platform services after developing the necessary Helm charts. The interactions between the deployed components are depicted in Figure 6. It is noted that we used the Nginx Kubernetes Ingress Controller as a reverse proxy to route incoming traffic towards the appropriate backend service. Table 4 summarizes the software version as well as the best-performing configuration for each software component according to our extensive preliminary experimentation.

### 5.2. Test Plan

In order to quantify the performance of our platform, we measured (i) the latency of the device data update requests (HTTP PATCH requests—they carry NGSI-based payloads) towards the REST API of the *Orchestrator* and (ii) the resource utilization (CPU and RAM utilization) for all platform services under a variable workload. For orchestrating the experiments, we created 10 different ecosystems and registered 10 IoT devices per ecosystem. Two different cases were examined, namely (i) ecosystems without IoT data integrity verification (no use of the *BC Service*) and (ii) ecosystems with IoT data integrity verification (use of the *BC Service*). Each data update request has a payload of 190 bytes. Furthermore, in order to evaluate the horizontal scalability of the platform, we leveraged the native Kubernetes horizontal scaling mechanism and created load-balanced replicas for the most resource-intensive services (*Orchestrator*, QuantumLeap, and Orion Context Data Broker), as observed during preliminary experimentation.

#### 5.2.1. Ecosystems without Data Integrity Verification

**Figure 7:**
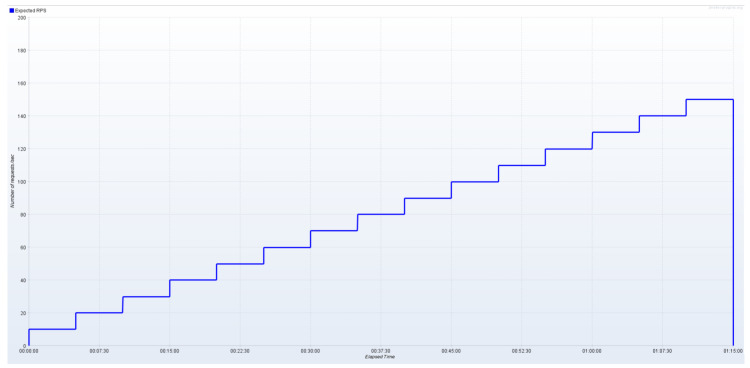
Step-wise increase in IoT data update load (no data integrity verification).

We initially increased the number of concurrent users (i.e., active JMeter threads) in a step-wise fashion in order to empirically quantify the maximum rate of requests/s the corresponding endpoint can successfully serve. This process was repeated independently for each number of replicas. Subsequently, we configured JMeter so as to generate asynchronous update requests and increase load in a step-wise fashion (step = 10 requests/s, up to the empirically measured maximum rate. Figure 7 depicts the corresponding test plan for a single replica per service. We summarize the parameters used in our experiments in Table 5.

#### 5.2.2. Ecosystems with Data Integrity Verification

**Figure 8:**
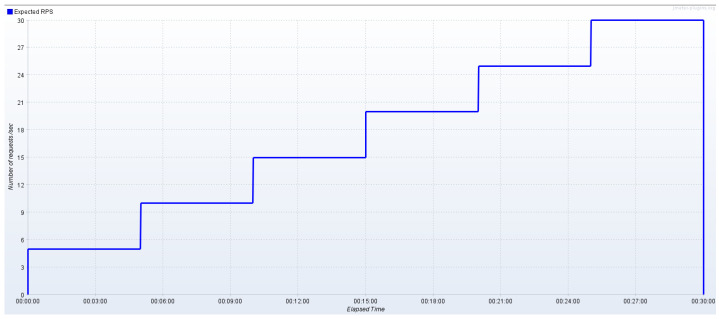
Step-wise increase in IoT data update load (with data integrity verification).

In this scenario, we utilized the *BC Service* through the Pearl BC Manager for implementing the IoT data integrity verification mechanism. Following the same strategy as described above, we initially increased the number of active JMeter threads in a step-wise fashion in order to empirically quantify the maximum rate of requests/s the corresponding endpoint can successfully serve. In this case, data update requests are served at a lower rate, mainly due to the consensus mechanism and the transaction processing overhead the *BC Service* introduces. As a result, we configured JMeter to generate asynchronous update requests by applying increasing load at levels (5–30 requests/s, step = 5), as shown in Figure 8. Having identified the communication with the *BC Service* as the bottleneck of this scenario, we solely use a single replica of any backend service.

### 5.3. Evaluation Results

In this section, we present the evaluation results for different IoT data update load levels. We provide the average and standard error (in the form of error bars) for CPU and RAM utilization of all backend platform services, each one running in separate (possibly replicated) Kubernetes pods, as well as the latency of IoT data update requests.

#### 5.3.1. Ecosystems without Data Integrity Verification

**Figure 9:**
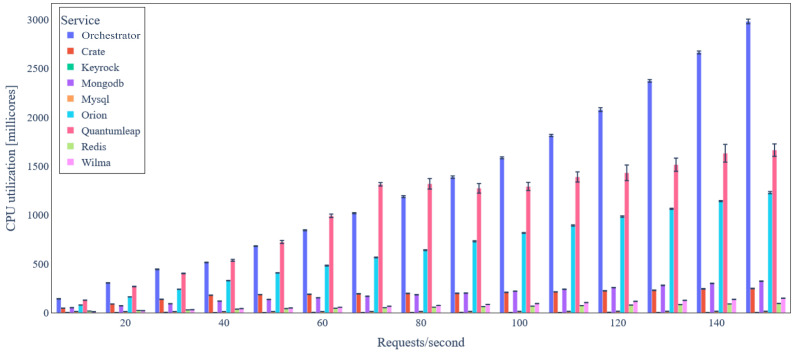
Backend service average CPU utilization under variable IoT data update request rate (basic scenario).

**Figure 10:**
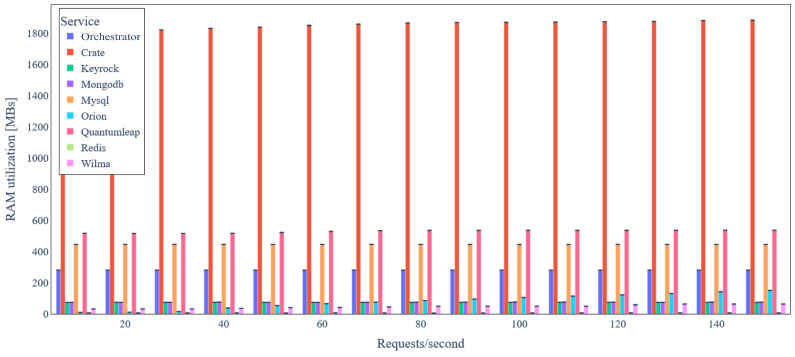
Backend service average RAM utilization under variable IoT data update request rate (basic scenario).

The average CPU and RAM utilizations of the backend services (each one corresponding to a unique Kubernetes pod replica) when no data integrity verification is applied are depicted in Figure 9 and Figure 10, respectively. As expected, average CPU utilization increases as data update rate increases for all backend services. Keyrock and MySQL services have the lowest CPU utilization, which is almost constant irrespective of the number of requests/s. This happens mainly due to the Wilma PEP Proxy caching of access control decisions that significantly reduces the number of requests towards the PDP resting in Keyrock. The two backend services with the highest average CPU utilization are the *Orchestrator* (3000 millicores for 150 requests/s) and QuantumLeap (1700 millicores fro 150 requests/s), both of them developed as web services using the Python Flask framework and Gunicorn WSGI HTTP server. For any service apart from the *Orchestrator*, CPU utilization increases less than linearly to the load (in requests/s), illustrating the scalability of the platform.

**Figure 11:**
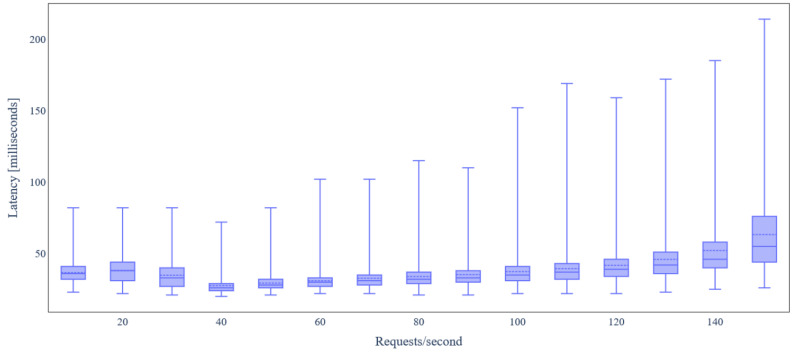
Latency under variable IoT data update request rate (basic scenario).

By carefully inspecting Figure 10, we conclude that most of the backend services have relatively low RAM requirements. CrateDB is the only service that requires almost 1.8 GB RAM, allocated as Java heap memory. In addition, there is a negligible increase in RAM utilization, as load increases. Figure 11 depicts the latency for IoT data update requests under varying load. We observe that the latency median remains almost constant and lower than 40ms in any case, except for 150 requests/s, where it is almost 55 ms.

**Figure 12:**
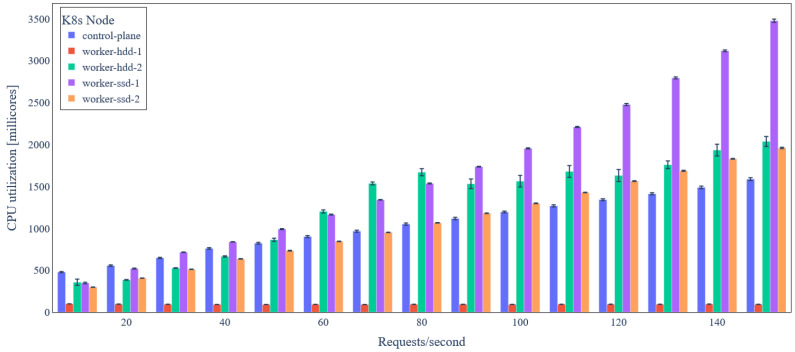
Average node CPU utilization under variable IoT data update request rate (basic scenario).

Figure 12 illustrates the average CPU utilization per node of the Kubernetes cluster the backend services are deployed on for the basic scenario (one replica per service). Observe that node “worker-ssd-1” utilizes almost 90% of the available CPU, while other worker nodes are underutilized. Based on this observation, as mentioned before, we leverage the Kubernetes horizontal pod scaling functionality for horizontally scaling the three most intensive backend services (*Orchestrator*, QuantumLeap, and Orion Context Data Broker) in order to improve utilization of available cluster resources. As shown in Table 5, two replicas may serve at most 190 requests/s (+27% compared to basic scenario), three replicas at most 240 requests/s (+60% compared to basic scenario), and four replicas at most 260 requests/s (+73% compared to basic scenario).

**Figure 13:**
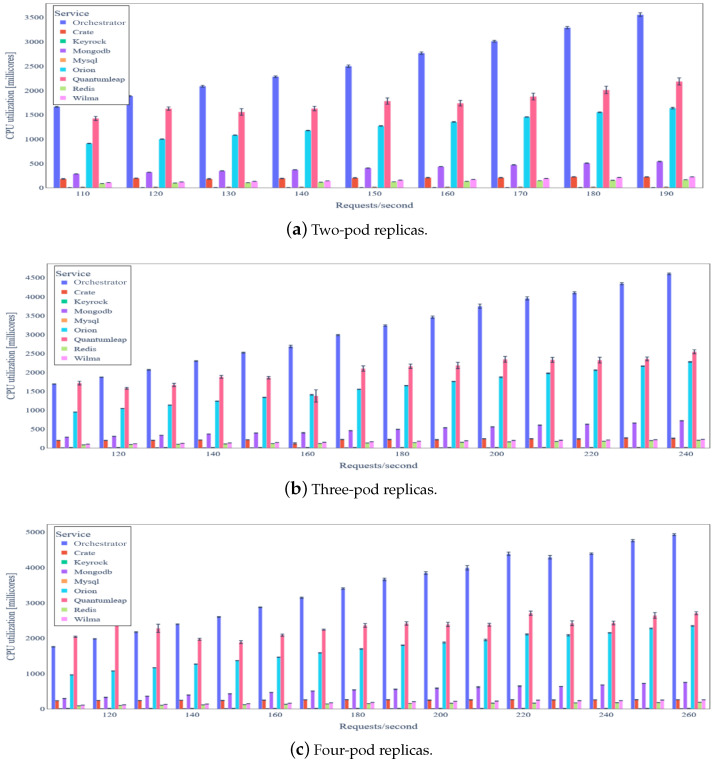
Backend services average CPU utilization under variable IoT data update request rate and horizontal scaling of three most intensive services.

Figure 13 illustrates the average CPU utilization of all backend services when the three most intensive services, namely *Orchestrator*, QuantumLeap, and Orion Context Data Broker, are horizontally scaled (two, three, and four pod replicas per service). For the sake of clarity, we omit rates lower than 100 requests/s, since average CPU utilization exhibits no significant differences compared to the basic scenario for these rates. We note that we report aggregate average CPU utilization per service by calculating the sum of the CPU utilization of all replicas. As in the basic scenario, average CPU utilization increases with the increase in the IoT data update rate. This happens in a sub-proportional manner for all services apart from the *Orchestrator*, possibly due to the worker model employed by the *Gunicorn* (https://gunicorn.org (accessed on 25 March 2024)) WSGI HTTP Server. In any case, the three backend services that are horizontally scaled remain the most CPU-intensive ones.

**Figure 14:**
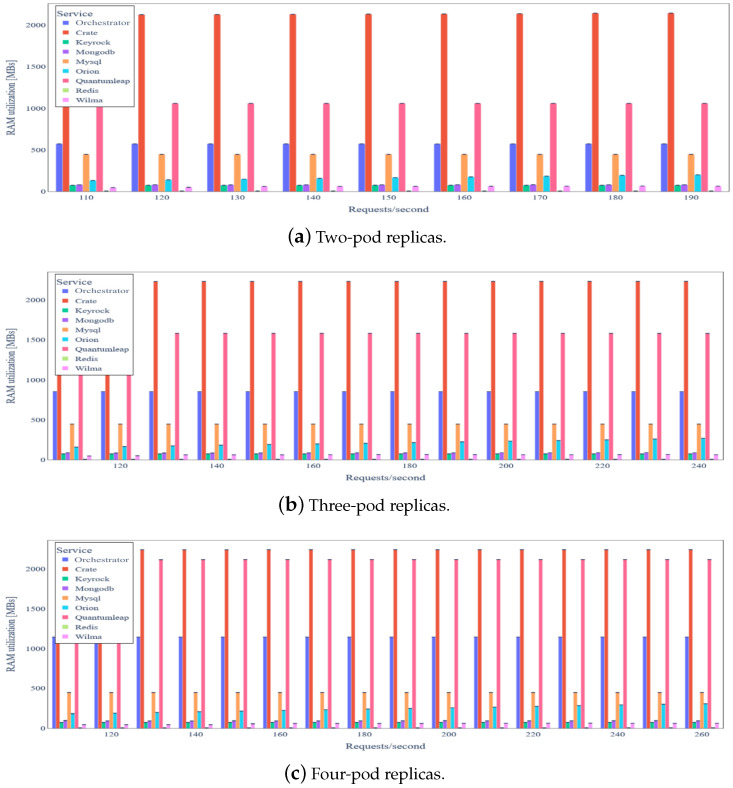
Backend service average RAM utilization under variable IoT data update request rate and horizontal scaling of three most intensive services.

The average RAM utilization for two, three, and four pod replicas of the three most intensive services is depicted in Figure 14. Similar to the basic scenario, CrateDB exhibits the highest RAM utilization (around 2.2 GB). RAM utilization for the *Orchestrator* and QuantumLeap services increases almost proportionally to the number of replicas. On the contrary, the Orion Context Data Broker’s RAM utilization is sub-proportional to the load, possibly due to more efficient memory management (application written in C++).

Table 6 summarizes the results in terms of resource utilization for a data update request rate of 150 requests/s, which is the maximum rate achieved for the baseline scenario. We observe small variations in terms of the aggregate CPU utilization for the three most CPU-intensive backend services. We still observe that the RAM utilization of the Orchestrator and QuantumLeap services rises nearly proportionally with the number of replicas, while that of the Orion Context Data Broker does not increase as much with the load, indicating a more efficient implementation of the latest. As expected, horizontal scaling of the three aforementioned backend services does not affect the utilization of any other backend service. Once more, the results indicate the fact that our system can scale efficiently with regard to the number of backend service instances.

**Figure 15:**
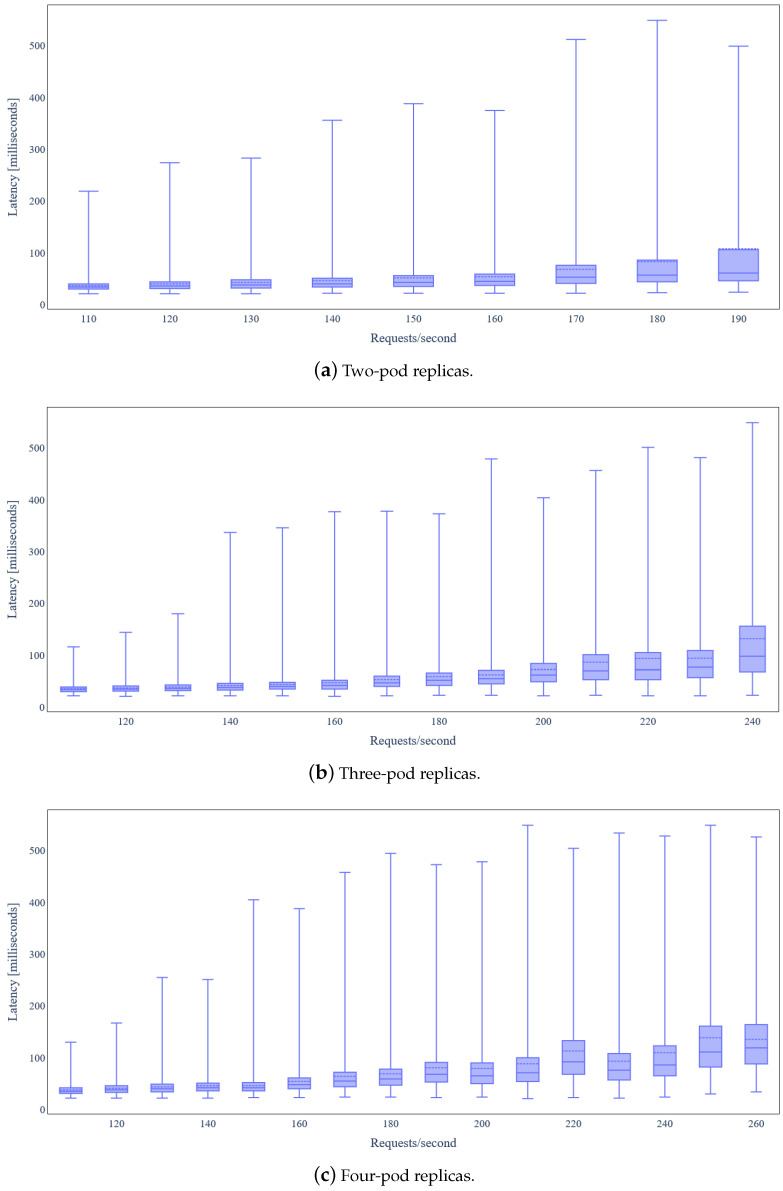
Latency under variable IoT data update request rate and horizontal scaling of the three most intensive services.

Finally, Figure 15 illustrates the latency of the IoT data update requests for horizontal scaling of the three most intensive services. The median latency is lower than 45 ms up to 150 requests/s but then increases more profoundly with the load increase, indicating increased pressure on the platform’s endpoint. In any case, the median latency is lower than 100 ms.

#### 5.3.2. Ecosystems with Data Integrity Verification

**Figure 16:**
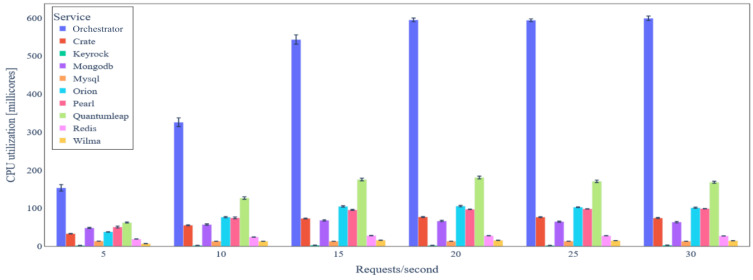
Backend service average CPU utilization under variable IoT data update request rate (with data integrity verification).

Here, we utilize the *BC Service* for the data integrity verification mechanism. Figure 16 illustrates the average CPU utilization of all backend services under variable IoT data update load in the case of data integrity verification. CPU utilization increases as the data update rate increases, up to the value of 20 requests/s. Then, CPU utilization remains almost constant due to the bottleneck introduced by the consensus mechanism of the *BC Service*. As previously, the services with the highest CPU utilization are the *Orchestrator* (600 millicores for 30 requests/s), QuantumLeap (170 millicores for 30 requests/s), and Orion Context Data Broker (105 millicores for 30 requests/s), but CPU utilization is in general considerably lower when compared to the one without data integrity verification.

**Figure 17:**
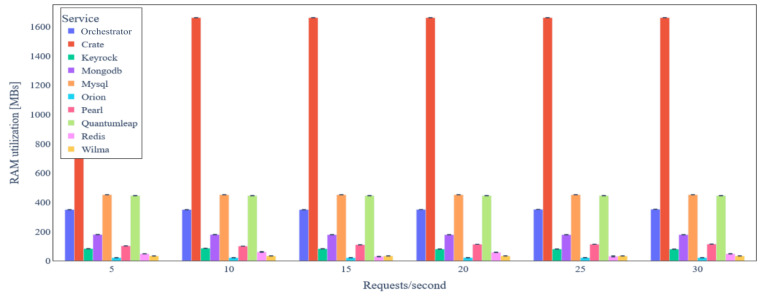
Backend service average RAM utilization under variable IoT data update request rate (with data integrity verification).

**Figure 18:**
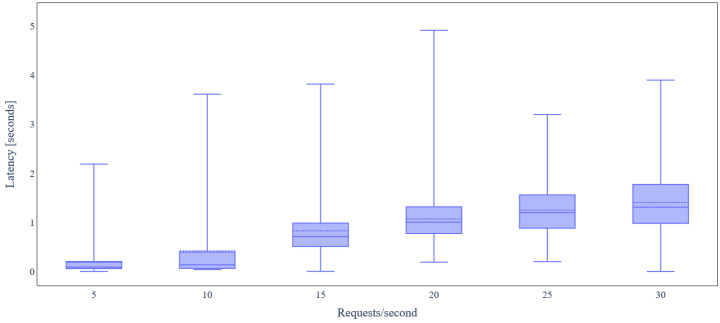
Latency under variable IoT data update request rate (with data integrity verification).

The average RAM utilization of the backend services when data integrity verification takes place is shown in Figure 17. No significant change in RAM utilization is observed as load varies. CrateDB still has the highest RAM demand (around 1.6 GB). Finally, Figure 18 depicts the latency for IoT data update requests under varying load when data integrity verification is performed. It is obvious that the median of the latency is significantly higher compared to the case where no data integrity verification takes place due to the delay of the consensus and transaction commit mechanisms introduced by the *BC Service*.

## 6. Conclusions and Further Work

In this paper, we proposed a Monitoring-as-a-Service platform for IoT applications based on FIWARE, which utilizes BC and SC technologies for data integrity verification. We presented the system architecture and thoroughly described the implementation details of our platform as well as the interactions between the system components. Additionally, we extensively evaluated a Proof-of-Concept Kubernetes-based deployment of the platform in terms of resource utilization (CPU, RAM) and latency under a variable rate of incoming IoT data. Most backend services, deployed as separate pods, have low computational requirements apart from three services that are more CPU intensive. By leveraging the native Kubernetes horizontal scaling functionality for the most intensive services, we achieve higher system throughput with a low expense in terms of RAM utilization. The evaluation shows that our platform enjoys scalability, provided that sufficient computational and memory resources are available. The incorporation of a BC-based data verification mechanism upheld the integrity of stored IoT data with no significant penalty on CPU and RAM utilization but at a discernible expense to the overall system throughput. Further work includes the investigation of Directly Acyclic Graph (DAG) ledgers such as IOTA (https://www.iota.org (accessed on 25 March 2024)) and NANO (https://nano.org (accessed on 25 March 2024)), which could support a much higher throughput in decentralized systems because the transactions can be sent and confirmed in parallel without the necessity to be grouped into sequential blocks as in traditional BC systems. Moreover, we aim to decentralize the identity management, authentication, and authorization mechanisms by substituting FIWARE Keyrock functionalities for corresponding processes that execute within SCs and use decentralized identifiers (https://www.w3.org/TR/did-core (accessed on 25 March 2024)) for enhanced privacy.

## Figures and Tables

**Table 1:** Related contributions that do not utilize BC technology.

Contribution	Scope	Commercial
City-level IoT [19]	Generic	No
MEWiN [20]	Agricultural water management	No
[21]	Health monitoring	No
[22]	Remote patient monitoring	No
SmartPort [23]	Seaport data monitoring	No
[24]	Energy management	No
Openhab [25]	Smart home applications	No
SmartThings [26]	Smart home applications	Yes
Apple HomeKit [27]	Smart home applications	Yes
Amazon Web Services IoT [28]	Generic	Yes
IBM Watson [29]	Generic	Yes

**Table 2:** Related contributions that utilize BC technology.

Contribution	Scope	Limitations
[30]	Generic	Standalone service not evaluated within a real platform; BC-compliant client required; fees have to be paid; significant overhead for encryption/decryption; whole data records stored in the public ledger
[31]	Generic	Data stored in BC; simulated evaluation system
[32]	Generic	Integrity verification process not supported by SCs
[33]	Generic	Resource-intensive cryptographic operations required for the clients; simulated BC used
[34]	Generic	Gas required for the SC execution; not clear which BC is used for the core functionalities; only data owners can retrieve data, thus there is no support for authorized third-party applications; this a standalone verification service not integrated within a real platform
[35]	Smart homes	Limited evaluation results provided; a private bitcoin network is used for BC that cannot support a high number of transactions; no flexibility is provided as SCs are not used; the scheme is not integrated or tested with a real platform
[36]	Smart cities	Increased latency due to multiple consensus algorithms’ execution; IoT devices required to send BC transactions

**Table 3:** Specifications of VMs deployed for performance evaluation.

	vCPUs	RAM	Storage	Operating System
**LG infrastructure**	4	8 GB	20 GB HDD	Ubuntu 20.04LTS
**CI infrastructure—** **master node**	4	10 GB	30 GB HDD	Ubuntu 20.04LTS
**CI infrastructure—** **worker nodes (4×)**	4	10 GB	30 GB HDD/SSD	Ubuntu 20.04LTS
**CI infrastructure—** **worker nodes (BC)**	4	16 GB	50 GB SSD	Ubuntu 20.04LTS

**Table 4:** Software versions and best-performing configuration for each software component.

Software Component	Software Version	Best Performing Configuration
MicroK8s	1.26.1	-
Orchestrator	0.1.0	• Gunicorn worker type: sync
		• Gunicorn workers: 9
Redis	7.0.7	-
Keyrock	8.0.0	-
MySQL	8.0.32	-
Orion	3.6.0	• reqMutexPolicy: none
		• reqPool: 4
MongoDB	4.4.11	-
QuantumLeap	0.8.0	• Gunicorn worker type: gthread
		• Gunicorn workers: 9
CrateDB	4.6.7	• Heap size: 2 GB
Wilma	8.0.0	-
Pearl	0.1.0	-
Hyperledger Fabric	2.4	-

**Table 5:** Experimental parameters (no data integrity verification).

Parameter	Values
Average data update load (Basic scenario—1 replica)	10–150 requests/s (step = 10)
Average data update load (2 replicas)	10–190 requests/s (step = 10)
Average data update load (3 replicas)	10–240 requests/s (step = 10)
Average data update load (4 replicas)	10–260 requests/s (step = 10)
Payload size	190 bytes

**Table 6:** Average CPU (milliCores) and RAM (MBs) utilization for data update request rate of 150 requests/s.

Service	Baseline		Two Pods		Three Pods		Four Pods	
	**CPU**	**RAM**	**CPU**	**RAM**	**CPU**	**RAM**	**CPU**	**RAM**
Orchestrator	2999	281	2497	572	2510	850	2593	1144
QuantumLeap	1660	537	1650	1058	1720	1583	1750	2102
Orion	1235	154	1230	167	1320	190	1361	212
MongoDB	352	75	346	80	353	85	367	89
Crate	266	1808	201	2130	215	2201	235	2230
Wilma	147	62	150	62	142	62	146	58
Redis	114	6	119	6	115	7	116	6
MySQL	15	446	14	445	15	446	15	446
Keyrock	2	74	2	75	3	75	3	74

## Data Availability

The data presented in this study are available on request from the corresponding author.

## Abbreviations

The following abbreviations are used in this manuscript:

API	Application Programming Interface
BC	Blockchain
CI	Cloud Infrastructure
CIM	Context Information Model
CPU	Central Processing Unit
CSS	Cloud Storage Service
DNS	Domain Name System
FTP	File Transfer Protocol
HLF	HyperLedger Fabric
HTTP	Hypertext Transfer Protocol
IoT	Internet of Things
LG	Load Generator
MaaS	Monitoring as a Service
MQTT	Message Queuing Telemetry Transport
NGSI	Next-Generation Service Interface
PDP	Policy Decision Point
PEP	Policy Enforcement Point
RAM	Random Access Memory
REST	Representational State Transfer
SC	Smart Contract
TPA	Third-Party Auditor
VM	Virtual Machine
